# High-Resolution Respirometry Reveals MPP^+^ Mitochondrial Toxicity Mechanism in a Cellular Model of Parkinson’s Disease

**DOI:** 10.3390/ijms21217809

**Published:** 2020-10-22

**Authors:** Pierpaolo Risiglione, Loredana Leggio, Salvatore A. M. Cubisino, Simona Reina, Greta Paternò, Bianca Marchetti, Andrea Magrì, Nunzio Iraci, Angela Messina

**Affiliations:** 1 Department of Biomedical and Biotechnological Sciences, University of Catania, V.le Andrea Doria 6, 95125 Catania, Italy; pierpaolo.risiglione@phd.unict.it (P.R.); salvatore.cubisino@phd.unict.it (S.A.M.C.); 2 Department of Biomedical and Biotechnological Sciences, University of Catania, Torre Biologica, Via Santa Sofia 97, 95125 Catania, Italy; loredanaleggio@unict.it (L.L.); greta.paterno.gp@gmail.com (G.P.); biancamarchetti@libero.it (B.M.); nunzio.iraci@unict.it (N.I.); 3 Department of Biological, Geological and Environmental Sciences, University of Catania, V.le Andrea Doria 6, 95125 Catania, Italy; simona.reina@unict.it; 4 we.MitoBiotech S.R.L., C.so Italia 172, 95125 Catania, Italy; 5 Neuropharmacology Section, OASI Research Institute-IRCCS, 94018 Troina (EN), Italy

**Keywords:** MPP^+^, mitochondria, Parkinson’s disease, high-resolution respirometry, SH-SY5Y cells

## Abstract

MPP^+^ is the active metabolite of MPTP, a molecule structurally similar to the herbicide Paraquat, known to injure the dopaminergic neurons of the nigrostriatal system in Parkinson’s disease models. Within the cells, MPP^+^ accumulates in mitochondria where it inhibits complex I of the electron transport chain, resulting in ATP depletion and neuronal impairment/death. So far, MPP^+^ is recognized as a valuable tool to mimic dopaminergic degeneration in various cell lines. However, despite a large number of studies, a detailed characterization of mitochondrial respiration in neuronal cells upon MPP^+^ treatment is still missing. By using high-resolution respirometry, we deeply investigated oxygen consumption related to each respiratory state in differentiated neuroblastoma cells exposed to the neurotoxin. Our results indicated the presence of extended mitochondrial damage at the inner membrane level, supported by increased LEAK respiration, and a drastic drop in oxygen flow devoted to ADP phosphorylation in respirometry measurements. Furthermore, prior to complex I inhibition, an enhancement of complex II activity was observed, suggesting the occurrence of some compensatory effect. Overall our findings provide a mechanistic insight on the mitochondrial toxicity mediated by MPP^+^, relevant for the standardization of studies that employ this neurotoxin as a disease model.

## 1. Introduction

Parkinson’s disease (PD) is a progressive age-related neurodegenerative disorder (ND) whose symptoms include motor system faults, such as resting tremors, rigidity and bradykinesia, and cognitive dysfunctions. These symptoms are the result of dopaminergic (DAergic) neurons loss within the *substantia nigra pars compacta* in the ventral midbrain and their terminal in the striatum, which lead consequently to striatal dopamine (DA) depletion [1]. The appearance of big spherical intraneuronal inclusions in the brainstem and cortex, called Lewy bodies (LBs) that contain protein aggregates in which α-synuclein (α-syn) is the main structural component, is one of the most common histopathological PD features [2]. Neuroinflammation is another important hallmark of the disease. In this context, the glial compartment—astrocytes and microglia—are pivotal in PD onset and progression. Notably, depending on the signals released in the microenvironment, glia may have a dual role, either beneficial or detrimental for DAergic neurons and neural stem cells exposed to harmful stimuli [3].

As for the other NDs, the cause(s) of PD are ill-defined and currently, there is no cure to stop or reverse PD progression [4]. The transplantation of relevant cell types represents a promising therapeutic strategy. On the other hand, new cell-free approaches, such as those based on extracellular vesicles, are emerging as innovative nanotherapeutics [5,6]. In this context, the definition at the molecular level of toxicity mechanisms in PD is crucial to support the development of potential therapeutic avenues.

To date, about 90 genes were linked to familial PD onset [7]. Despite this, the etiology of the vast majority (up to 90%) of so-called “idiopathic” cases is multifactorial, recognized to arise from a combination of polygenic inheritance and environmental exposures [8,9]. Notably, a wide panel of environmental factors including neurotoxicants, commonly used as herbicides or pesticides, have long been recognized as critical risk factors for PD [10].

MPTP (1-methyl-4-phenyl-1,2,3,6-tetrahydropyridine) is a molecule structurally similar to the herbicide Paraquat and was the first neurotoxicant shown to induce in humans a profound parkinsonian syndrome [11,12]. MPTP injures, in a selective manner, the dopaminergic neurons in the nigrostriatal system, and when tested in various animal species, including non-human primates, it showed the ability to recapitulate most PD-like symptoms [13,14]: i.e., the long exposure to low MPTP doses promotes the increase of oxidative stress, α-syn fibrillization, and loss of mitochondrial functionality [15,16,17]. Since MPTP is a highly lipophilic compound, it rapidly crosses the blood–brain barrier and after systemic exposure, the toxin levels are already detectable in the brain within minutes. By itself, MPTP is not a toxic substance, however, once in the brain, it is metabolized to 1-methyl-4-phenyl-2,3-dihydropyridinium (MPDP) by the enzyme monoamine oxidase B (MAO-B) in non-dopaminergic cells (i.e., the astrocytes) [18]. Next, MPDP is oxidized to the active 1-methyl-4-phenylpyridinium (MPP^+^) which is then released into the extracellular space, where it is taken up by the dopamine transporter (DAT) and is concentrated within the dopaminergic neurons, causing the specific loss of nigrostriatal neurons [19,20,21].

Within the cells, MPP^+^ exerts its toxicity gathering in mitochondria, where it blocks the activity of NADH-ubiquinone oxidoreductase (complex I) of the mitochondrial electron transport system (ETS), leading to ATP depletion and reactive oxygen species (ROS) production [22,23,24]. Moreover, the prolonged exposure to the toxin causes a drastic inhibition of mtDNA-encoded respiratory subunits synthesis [25]. Despite these events having long been thought to be strictly related to DAergic neuronal loss in PD, another report suggests that neuronal death induced by MPP^+^ is independent from complex I inhibition [26].

Neuroblastoma SH-SY5Y cells exposed to MPP^+^ are one of the most used in vitro models in PD research. These cells, deriving from the SK-N-SH line, show a moderate activity of tyrosine hydroxylase (TH) and dopamine-β-hydroxylase, conferring to the line a catecholaminergic phenotype [27,28]. SH-SY5Y cells may be differentiated in order to induce a “more-neuronal” phenotype: the addition of retinoic acid (RA) to the cell culture medium, in a concentration ranging from 5 to 20 µM, represents the best approach [29]. The differentiation time may vary from 1 to 21 days during which cells switch toward a neuronal and DAergic phenotype [29]. All these variations found in protocols for SH-SY5Y culture and differentiation possibly contribute to the different functional outcomes [30]: for instance, in specific circumstances, differentiation treatment increases susceptibility to neurotoxins exposure [31], while in others no change or even a decrease was observed [32].

The large use of this in vitro model, however, was not accompanied in the years by a detailed characterization of the mitochondrial respiration. By delivering MPP^+^ to differentiated SH-SY5Y cells, in this work, we performed an in-depth analysis of oxygen flows corresponding to each respiratory state using high-resolution respirometry (HRR). Beyond an expected slight decrease in complex I activity, an accentuated raise of the dissipative component of respiration was observed, indicating the presence of extended damage in the inner mitochondrial membrane (IMM). Moreover, we uncovered a previously unknown compensatory effect of succinate dehydrogenase (complex II) as a response to the partial block of complex I activity exerted by toxin treatment. Overall, our findings depict a more intricate mechanism of MPP^+^ mitochondrial toxicity.

## 2. Results

### 2.1. MPP^+^ Toxicity Assessment in Differentiated SH-SY5Y Cells

**Figure 1:**
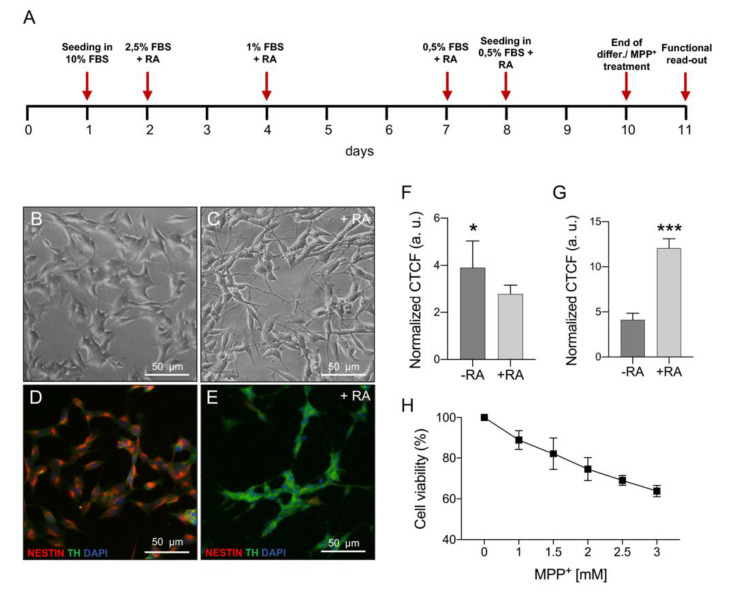
Phenotypic characterization of undifferentiated vs. differentiated SH-SY5Y cells and MPP^+^ dose–response curve. (**A**) Timeline of the differentiation protocol. (**B,C**) Brightfield images showing morphological differences between undifferentiated cells on day zero (**B**) and differentiated cells at day nine (**C**); RA, retinoic acid. (**D,E**) Immunofluorescence images showing the different expression of nestin (red) and TH (green) in undifferentiated (**D**) and differentiated (**E**) cells. (**F,G**) Relative quantification of nestin (**F**) and TH (**G**) in undifferentiated and differentiated cells. (**H**) Dose–response curve for selected MPP^+^ concentrations on differentiated SH-SY5Y cells. * *p* < 0.05 and *** *p* < 0.001.

Neuroblastoma SH-SY5Y cells were differentiated with RA and serum reduction for nine days prior to MPP^+^ treatment, using the protocol schematized in Figure 1A. The differentiation induced morphological changes as highlighted in the brightfield and immunofluorescence images. In comparison with undifferentiated cells, RA-differentiated SH-SY5Y displayed thin and branching neurites usually resulting in a network formation (Figure 1B,C) [33]. Moreover, immunofluorescence analysis on RA-treated cells showed a decreased expression of the non-differentiated cell marker nestin (Figure 1D,F for quantification) and a higher expression of TH (Figure 1E,G for quantification), which plays a central role in DA synthesis [31]. In order to evaluate MPP^+^ mitochondrial toxicity on a DAergic model, RA-differentiated SH-SY5Y cells were exposed to increasing toxin concentration and cell viability was determined after 24 h, performing a dose–response curve. As shown in Figure 1H, cell viability varied from 89% to 64% ranging from 1 to 3 mM of MPP^+^. Based on this result, we selected the lowest MPP^+^ dose for further experiments assuming that, in this condition, any mitochondrial impairment is not ascribed to a consistent cell death but rather to the direct organelle damage caused by the neurotoxin.

### 2.2. MPP^+^ Drastically Reduces Oxygen Consumption Associated with the Main Respiratory States

**Figure 2:**
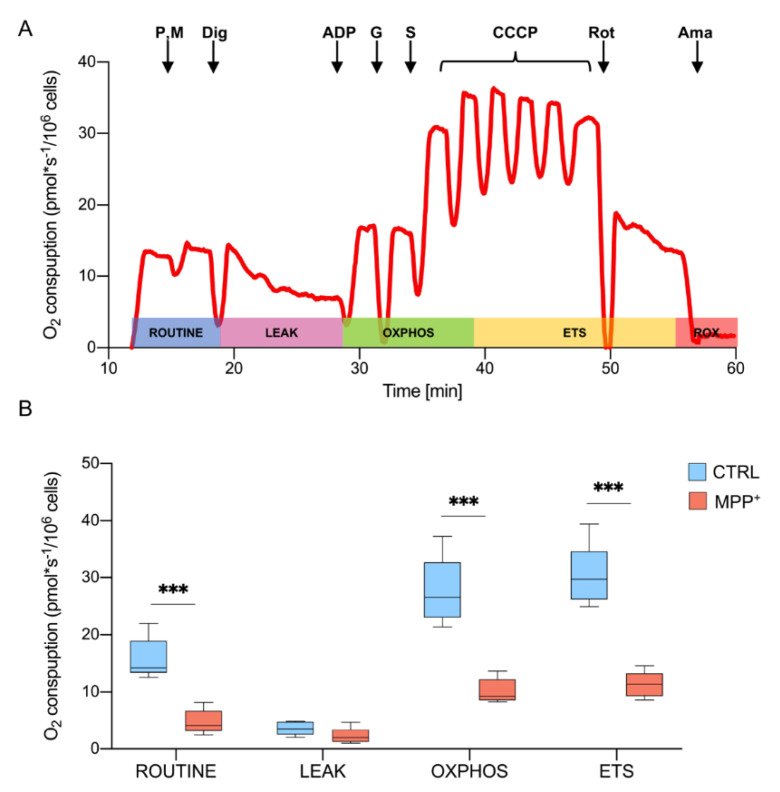
Oxygen consumption in differentiated SH-SY5Y cells. (**A**) The representative curve of mitochondrial respiratory function of untreated RA-differentiated SH-SY5Y cells assayed in MiR05 respiration medium at 37 °C. The respiratory states ROUTINE, LEAK, OXPHOS, ETS, and ROX were analyzed after the addition of specific substrates and inhibitors. P, pyruvate; M, malate; Dig, digitonin; G, glutamate; S, succinate; Rot, rotenone; Ama, antimycin. (**B**) Quantitative analysis of the oxygen consumption rate expressed as pmol/second per million cells of control and MPP^+^ treated cells calculated for ROUTINE, LEAK, OXPHOS, ETS. Data are shown as mean with standard deviation, with *** *p* < 0.001.

The oxygen consumption profile of RA-differentiated SH-SY5Y cells was analyzed by HRR using a specific substrate-uncoupler-inhibitor titration (SUIT) protocol. This protocol allows the investigation of the main respiratory states exploiting both endogenous and externally added substrates, that reached mitochondria after cell permeabilization [34]. In Figure 2A, a representative trace of oxygen consumption of untreated cells (control) illustrates the SUIT protocol. Briefly, the physiological oxygen consumption, corresponding to ROUTINE state, was measured in intact cells. Then, endogenous substrates (including ADP) were forced to leave the cells by permeabilization of the plasma membrane. Therefore, the remaining oxygen consumption was related to the non-phosphorylating or LEAK state. The OXPHOS state, indicating the oxygen flow sustained by the mitochondrial respiratory chain, was achieved in the presence of pyruvate, malate, glutamate, succinate, and ADP, the last in saturating concentration. Next, the maximal respiratory ETS capacity was obtained at the optimal uncoupler concentration. Finally, the specific inhibition of complex I and III with rotenone and antimycin respectively, allowed for the determination of the residual respiration or ROX [34,35].

Oxygen consumption, corresponding to each respiratory state in cells treated with 1 mM MPP^+^ for 24 h, was monitored and compared to the control. The obtained values were corrected for the ROX and are reported in Figure 2B (see Supplementary Materials Table S1 for raw data). As shown, a dramatic reduction of oxygen flow was detected in ROUTINE (−70%, *p* = 0.0004, *n* = 5), OXPHOS (−70%, *p* = 0.0003, *n* = 5) and ETS (−62.7%, *p* < 0.0001, *n* = 5). Prior to a very moderate cell death, as previously observed in the same condition, these results indicate that MPP^+^ severely affects the respiratory capacity of mitochondria.

### 2.3. MPP^+^ Increases the Dissipative Ratio of Oxygen Flux

The oxygen consumption depends on a sum of different factors, including obviously ETS activity but also mitochondrial mass and biogenesis, all features subjected to change upon MPP^+^ exposure. For instance, MPP^+^ increases autophagy and mitochondria degradation in SH-SY5Y cells [36,37], affects organelle morphology, mass and protein expression in RA-differentiated SH-SY5Y [25], and the mitochondrial dynamic in vivo [38].

**Figure 3:**
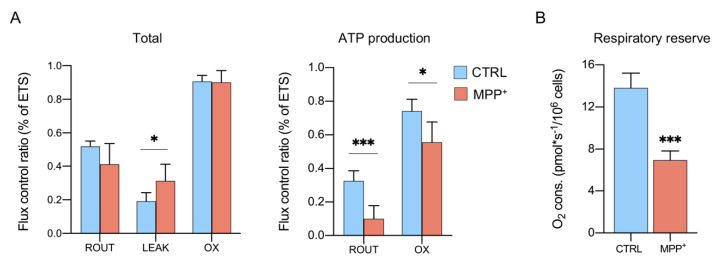
MPP^+^ effect on mitochondrial respiration. (**A**) Oxygen consumption measured in different respiratory states in the presence or not of MPP^+^, expressed as the total or ATP-related flux. Data are displayed as the flux control ratio (FCR) of the maximal ETS capacity. (**B**) Oxygen flux related to the respiratory reserve of cells in the presence or not of MPP^+^. All data are shown as means with standard deviation; * *p* < 0.05 and *** *p* < 0.001.

Flux control ratios (FCRs), instead, express respiratory states independently of mitochondrial content since they are obtained by normalizing each flux for the maximum flux [34,35,39]. In Figure 3A (see also Supplementary Materials Table S2), ROUTINE, LEAK, and OXPHOS respiration are indicated as the ratio of maximal ETS capacity. As shown in the left panel, MPP^+^ treatment affected specifically the LEAK state: a significant increase (+63%) was observed in treated cells (0.19 ± 0.05 in control vs. 0.31 ± 0.09 in treated cells, *p* = 0.046, *n* = 5), while no significant differences were detected for ROUTINE and OXPHOS respiration between the samples. Since the LEAK state represents the amount of dissipative flux, this result suggests the presence of some injury at the IMM level which impinges on the proton gradient maintenance and, in turn, on ATP production [40]. As reported in the right panel, the phosphorylation-related flux is severely affected in ROUTINE (−71.9%, *p* = 0.0009, *n* = 4) and in a less dramatic manner in OXPHOS (−18.3%, *p* = 0.0039, *n* = 4). Accordingly, the respiratory reserve (RR), consisting of the extra ATP produced by oxidative phosphorylation in case of an increased energy demand [41,42] was found more than halved in MPP^+^ treated cells (Figure 3B, −55%, *p* = 0.0002, *n* = 4) (see also Supplementary Materials Table S3).

Taken together, these results suggest that neurotoxin affects IMM integrity decreasing substantially the phosphorylation processes.

### 2.4. MPP^+^ Reduces Coupling Efficiency in Each Respiratory State

Coupling efficiency represents another important parameter to evaluate MPP^+^ effect on mitochondrial respiration. It indicates the ratio of oxygen flux coupled to ADP phosphorylation in a specific respiratory state [35].

**Figure 4:**
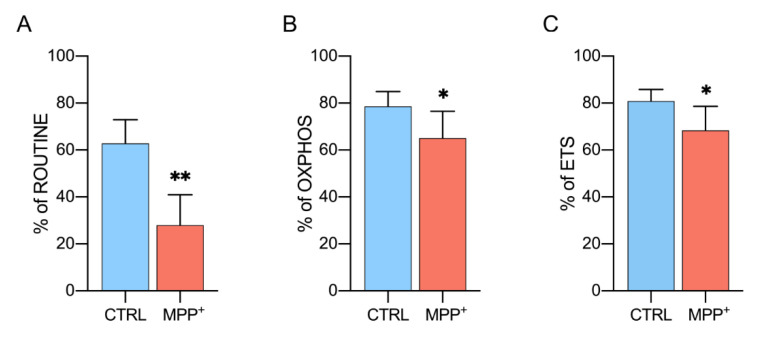
Coupling efficiency of different respiratory states. Rate of oxygen flux during ROUTINE (**A**), OXPHOS (**B**), and ETS (**C**) coupled with the ADP phosphorylation. Data are shown as the percentage of the reference state and expressed as means with standard deviation; * *p* < 0.05 and ** *p* < 0.01.

Figure 4A shows the coupling degree of the ROUTINE state. In control cells, about 63% of oxygen consumption is coupled with the phosphorylation process, while only 28% of the flux is coupled in MPP^+^ treated cells (−62.9%, *p* = 0.0026, *n* = 4). Similarly, the coupling efficiency of OXPHOS and ETS was significantly decreased upon neurotoxin exposure, even if in a less dramatic manner. In particular, phosphorylation-related oxygen flux decreased from 78% (control cells) to 65% (MPP^+^ treated) in OXPHOS (−16.7%, *p* = 0.0488, *n* = 4) and from 80% to 68% in ETS (−15%, *p* = 0.0426, *n* = 5) as reported in Figure 4B,C.

Overall, the observed reduction of coupling degree in all respiratory states confirms the strong impact of MPP^+^ on the phosphorylating flows previously described.

### 2.5. Activity of Complex II Is Increased Upon Complex I Inhibition

**Figure 5:**
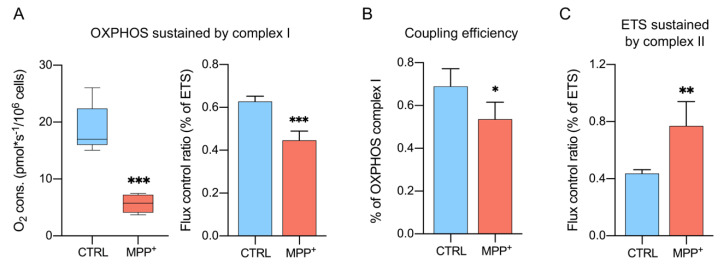
Specific contribution of complex I and complex II to different respiratory states. (**A**) Quantitative analysis of the oxygen consumption rate and the relative FCR of OXPHOS state sustained by complex I. Data were obtained by measuring OXPHOS respiration in the absence of succinate. (**B**) Rate of oxygen flux OXPHOS respiration sustained by complex I coupled with the ADP phosphorylation. (**C**) Quantitative analysis of the oxygen consumption rate and the relative FCR of ETS sustained by complex II. Data were obtained by measuring ETS after inhibition of complex I activity with rotenone. Data are shown as percentage of the reference state and expressed as means with standard deviation; * *p* < 0.05, ** *p* < 0.01, and *** *p* < 0.001.

Since NADH-ubiquinone oxidoreductase is a specific target of MPP^+^ [22], HRR was used to evaluate the contribution of this specific complex to the OXPHOS respiration. This was achieved in the presence of pyruvate, malate, glutamate, and ADP but not of succinate, which conversely stimulates complex II. Figure 5A shows both the oxygen consumption level and the corresponding FCRs. As occurred for the other respiratory states, the oxygen level of OXPHOS sustained by complex I was found substantially lowered after MPP^+^ addition (−63.1%, *p* = 0.0002, *n* = 5). Similarly, a moderate decline of FCR was observed (−18%, *p* = 0.0003, *n* = 4). Accordingly, the degree of ATP-coupled respiration sustained exclusively by complex I was found significantly reduced (Figure 5B), varying from 69% of the control to 53% of MPP^+^ treated cells (−23%, *p* = 0.043, *n* = 4).

After total OXPHOS stimulation and ETS measurement, complex I was inhibited by using rotenone. With ETS calculated in the presence of all substrates (including succinate), the measurement was related to the specific contribution of complex II to ETS. Despite a general reduction of oxygen flux in the presence of MPP^+^ (−38.3%, *p* = 0.0156, *n* = 5, Table S1), Figure 5C indicates a significant increase (+81%) in the FCR compared to control (*p* = 0.0084, *n* = 4, Table S2) suggesting the existence of some compensatory mechanism(s) put in place by succinate dehydrogenase as a response to complex I inhibition.

To further investigate this aspect and clarify whether a direct link between complex II and the toxin exists, the activity of succinate dehydrogenase was specifically assayed with a distinct HRR protocol aimed to evaluate the electron flow into the Q-junction occurring independently from complex I. It is indeed known that, when complex I is stimulated (as occurred in the protocol in Figure 2A), oxaloacetate rapidly accumulates within mitochondria, acting as a potent inhibitor of complex II, already at low doses [39,43].

**Figure 6:**
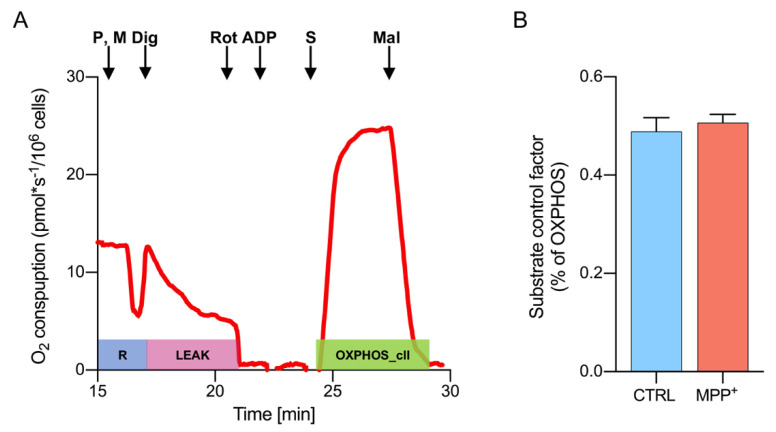
Activity of complex II assayed by HRR. (**A**) Representative curve of mitochondrial respiratory function of untreated RA-differentiated SH-SY5Y cells assayed in MiR05 respiration medium at 37 °C. The respiratory states ROUTINE, LEAK, and OXPHOS driven by complex II were obtained after the addition of specific substrates and inhibitors. P, pyruvate; M, malate; Dig, digitonin; Rot, rotenone; S, succinate; Mal, malonic acid. (**B**) Quantitative analysis of the oxygen consumption rate of control or MPP^+^ treated cells of OXPHOS sustained by complex II. Data are expressed as the percentage of the total OXPHOS and shown as SCRs (mean with standard deviation).

Figure 6A shows a representative oxygen consumption curve of untreated differentiated SH-SY5Y cells, along with the protocol used for complex II stimulation. Briefly, cells were permeabilized in the presence of pyruvate and malate but complex I was immediately inhibited by rotenone, thus avoiding oxaloacetate accumulation. Next, the addition of ADP did not induce any response, as demonstrated by the oxygen flow curve kept around zero (Figure 6A). Then, complex II was activated by succinate. As a counter-proof, the addition of the succinate dehydrogenase inhibitor malonic acid brought the oxygen curve back to zero. The complex II-linked oxygen flows were analyzed for control and MPP^+^ treated cells, normalizing values for the convergent complex I + complex II electron supply flows [35,39]. The results are displayed in Figure 6B as substrate control factor (SCF). As reported, no significant difference in complex II activity was observed between samples (0.49 ± 0.03 in control vs. 0.52 ± 0.01 in treated cells, *p* = 0.442, *n* = 3). These data indicate that the enhancement of succinate dehydrogenase activity, previously observed in MPP^+^ treated cells (Figure 5C) likely depends on the inhibition of complex I activity, rather than from the toxin itself.

## 3. Discussion

Mitochondrial dysfunction is an important hallmark of PD, together with protein aggregation and oxidative stress. These features, however, are common to all NDs and are strictly interconnected. Protein aggregates interact with the cytosolic surface of mitochondria and impair metabolic exchanges with the organelle. Aβ oligomers in Alzheimer’s disease [44,45], SOD1 mutants in Amyotrophic Lateral Sclerosis (ALS) [46,47,48], and α-syn in PD [49], all associate with the Voltage-Dependent Anion Channels (VDAC) isoform 1, reducing dramatically the mitochondria synthesized ATP availability [50]. VDACs are the most abundant mitochondrial porin, evolutionarily conserved from yeast to humans, playing a fundamental role for organelle physiology [51,52,53]. It has been recently demonstrated that a reduction of VDAC1 function affects mtDNA synthesis and, in turn, the expression of mitochondrial genes encoding for essential subunits of ETS enzymes [54]. Also, in PD models, VDAC1 favors α-syn translocation within mitochondria [55] where it is believed responsible for the impairment of complex I and IV activity [56,57]. Therefore, ETS damage represents a key event in mitochondrial dysfunction onset in PD.

Although, in a different way, the lipophilic cation MPP^+^ accumulates in mitochondria and inhibits the electron flow from complex I to coenzyme Q, contributing to ATP depletion and ROS increase [17,24,58]. For this reason, MPP^+^ is widely used for the induction of a PD-like phenotype in different in vitro models, such as the RA-differentiated SH-SY5Y cell line used herein, as a valid alternative to neuronal cells treatment with α-syn oligomers and fibrils, or to genetic models. Thus, it is crucial to understand the precise changes in mitochondrial respiration upon the MPP^+^ treatment. To this end, here we applied HRR technology highly sensitive and capable to deeply analyze oxygen consumption levels associated with each respiratory state and to ETS complexes.

We found that the treatment with 1 mM MPP^+^ on RA-differentiated cells resulted in a slight decrease of cell viability (~10%) in front of a drop of oxygen consumption associated with the main respiratory states, up to 70%. These data indicate that MPP^+^ exerts a specific mitochondrial toxicity, possibly depending on a reduction of both mitochondrial functionality and organelle mass [22,33,34]. Focusing on the specific differences within mitochondria, we performed a rigorous analysis of FCRs from which at least two intriguing results came out.

First, a significant increase of the dissipative flux, the LEAK state, was observed in cells upon MPP^+^ exposure. The LEAK state depends mainly on the IMM integrity and on proton and electron leaking. In the case of IMM injury, protons by-pass ATP synthases, promoting a decrease in ATP production, and electrons escape the ETS pathway, as they are addressed to other substrates, increasing thus ROS formation [59]. The rise in LEAK respiration indicates the presence of extensive IMM damage in MPP^+^ treated cells and this explains data in the literature about ATP depletion and oxidative stress induction [37,60]. Accordingly, oxygen flows devoted to ATP synthesis (the so-called net respiration) were found significantly decreased, drastically in ROUTINE and in a moderate manner during OXPHOS. This result was strengthened by a similar reduction in the coupling degree measured in all respiratory states.

**Figure 7:**
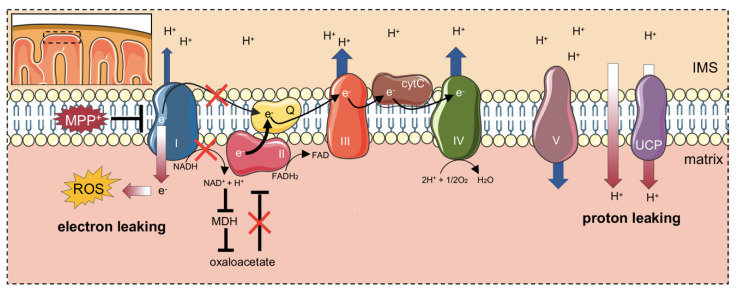
Proposed mechanism of MPP^+^ toxicity at the IMM level. MPP^+^ promotes the impairment of complex I activity. Electrons are then addressed towards other substrates, increasing ROS production (electron leaking). The reduced activity of complex I affects MDH activity as well as the accumulation of the Krebs cycle intermediates and complex II inhibitor oxaloacetate. In this perspective, the activity of complex II raises since it is less subjected to oxaloacetate inhibition. MPP^+^ also induces UCPs gene expression. The increased activity of UCP proteins dissipates partially the proton gradients (proton leaking), a mechanism called “mild uncoupling” and aimed to counteract ROS damage. Original figure (created with https://smart.servier.com tools).

Second, in the presence of MPP^+^ the contribution of complex I to the OXPHOS respiration was remarkable reduced, as expected. At the same time, a significant increase in complex II activity during ETS was detected, indicating a compensatory mechanism put in place by complex II as a response to the partial inhibition of NADH-ubiquinone oxidoreductase and/or to the toxin. However, the same effect was not as strong as when the complex II activity was tested in association with the complete inhibition of complex I. Nonetheless, this result is not so surprising. It is known that a reduction of complex I activity, as found in MPP^+^ treated cells, negatively impacts on malate dehydrogenase (MDH) activity, as a result of NAD^+^ to NADH redox shift, affecting in turn oxaloacetate synthesis [39]. Since oxaloacetate is a potent inhibitor/modulator of succinate dehydrogenase, especially in nervous tissues [43], it is reasonable to speculate that complex II is less subjected to oxaloacetate inhibition in MPP^+^ treated cells, due to the low activity of complex I. Notably, the main difference in the SUIT protocols applied here consists in the use of saturating rotenone concentration at different stages. Accordingly, in Figure 2A, rotenone was added after complex I stimulation, allowing oxaloacetate to accumulate, whereas in Figure 6A complex I was directly inhibited, in order to avoid any eventual increase of oxaloacetate concentration. Therefore, the higher activity of complex II observed in the Parkinson’s-like phenotype, i.e., where complex I is only partially inhibited by MPP^+^ (as in Figure 5C) and not completely with the saturating rotenone concentration (as technically required for Figure 6 experiments), likely represents a response to complex I inhibition, rather than a direct effect of the neurotoxin. Other studies showed results in agreement with those found here. For example, Calabria and colleagues have found a compensatory effect ascribed to complex II, as well as an increase in LEAK respiration in NSC-34 cells expressing the SOD1 G93A mutant, a model of ALS [61]. Notably, this was explained as a response to the decreased activity of complex I, exerted by the SOD1 mutant [61]. Taken together, these findings depict the existence of a common rescue mechanism put in place by complex II in response to different stimuli (toxins, protein aggregates) affecting complex I activity. Furthermore, these effects can be only partially ascribed to the direct complex I impairment promoted by MPP^+^. In fact, it is known that cells use LEAK respiration as a protective strategy under certain circumstances. IMM contains a group of transporters called uncoupling proteins (UCPs), whose function is to partially dissipate the mitochondrial membrane potential in the form of heat [62,63]. The inhibition of complex I increases oxygen radical formation that is counteracted by the dissipation of a part of hyperpolarized IMM potential, a process known as “mild uncoupling”, with the final aim to neutralize the ROS effect [64]. Not coincidentally, a time- and dose-dependent induction of UCP2, 4, and 5 expression in neuronal cells was observed after exposure to MPP^+^ [65]. Notably, UCP4 overexpression exerts a neuroprotective effect on SH-SY5Y upon MPP^+^ treatment and stimulates complex II activity by its direct interaction, thus promoting an ATP level increase [66,67,68]. A cartoon depicting our proposed model is displayed in Figure 7.

In any case, although moderate, the MPP^+^ dose regimen here applied in SH-SH5Y cells was higher compared to those generally used in the highly vulnerable primary dopaminergic neurons, that are severely injured upon exposure to ≤50 µM MPP^+^ or to the false neurotransmitter 6-hydroxydopamine [69]. Therefore, further studies are needed to disclose the herein described mitochondrial effects of MPP^+^ on primary mesencephalic neuronal cultures, that will be relevant for dopaminergic neuron physiopathology.

In conclusion, by using high-resolution respirometry tools we have identified and explained the bioenergetics fault and recovery found in MPP^+^ treated cells, where mitochondria complex I was inhibited. By a precise analysis, we demonstrated the relevance of complex II in this recovery mechanism. These results are relevant to understand the mitochondria dysfunction in PD, and possibly in other NDs.

## 4. Materials and Methods

### 4.1. Cell Culture and Differentiation

The neuroblastoma cell line SH-SY5Y was purchased from ICLC (Interlab Cell Line Collection, accession number ICLC HTL95013; obtained from depositor European Collection of Authenticated Cell Cultures (ECACC)) and maintained in MEM/F12 medium supplemented with 10% fetal bovine serum, 2 mM L-glutamine and 1% penicillin/streptomycin. For cell differentiation, the protocol was adapted from [31,70]. Briefly, MEM/F12 was replaced with DMEM/F12 and 10 µM all-trans RA (Sigma Aldrich, St. Louis, MO, USA) was added. The medium was changed every three days, lowering the serum amount to reach the final concentration of 0.5% by the seventh day. Cells were seeded at a density of 3 × 10^5^ cells/cm^2^ in 6, 24, or 96 well plates.

### 4.2. Immunofluorescence

Cells seeded on poly-L-lysine coated glass coverslips (Sigma Aldrich) were fixed after treatment with 4% paraformaldehyde and stained with primary mouse anti-nestin antibody (sc-23927, Santa Cruz, 1:50) and with rabbit anti-TH (Tyrosine hydroxylase) antibody (AB152, Sigma-Aldrich, 1:250). Nuclei were stained with DAPI (Merck, Kenilworth, NJ, USA). Donkey anti-mouse Alexa Fluor 546 or donkey anti-rabbit Alexa Fluor 488 secondary antibodies were used (Thermo Fisher, Waltham, MA, USA). Fluorescence intensity was quantified using ImageJ software. Values are indicated as normalized corrected total cell fluorescence (CTCF) expressed as arbitrary units (A.U.).

### 4.3. MPP^+^ Treatment and Cell Viability

Treatment with MPP^+^ (Sigma Aldrich) was performed on day 10 of the differentiation protocol. Cells seeded in 96-well black microplates were treated for 24 h with the reported final concentrations of neurotoxin. The dose–response curve was performed using the following doses: 1, 1.5, 2, 2.5, and 3 mM. Cell viability was determined using the Celltitre-Blue Cell Viability Assay (Promega, Madison, WI, USA) according to the manufacturers’ recommendations. Fluorescence intensity was measured using the microplate reader Varioskan (Thermo Scientific, Waltham, MA, USA).

### 4.4. High-Resolution Respirometry (HRR) Analysis

Mitochondrial respiration capacity of RA-differentiated SH-SY5Y was analyzed by HRR using the two-chamber system O2k-FluoRespirometer (Oroboros Instruments, Innsbruck, Austria). Cells were harvested, counted, and resuspended in the mitochondrial respiration buffer Mir05 (Oroboros Instrument). All the experiments were performed at 37 °C under constant stirring of 750 rpm. Oxygen consumption in the various respiratory states was determined using a SUIT modified protocol [31] and the contribution of specific mitochondrial complexes to respiratory capacity was investigated.

ROUTINE respiration was measured in intact cells. Permeabilization of the plasma membrane was achieved by using the mild detergent digitonin (Sigma Aldrich) at the final concentration of 4.07 μM. This concentration was previously determined in order to allow the access of substrates across the plasma membranes but without compromising mitochondrial membranes integrity. LEAK was measured after plasma membrane permeabilization and in the presence of previously added pyruvate and malate. A total of 5 mM pyruvate (P), 2 mM malate (M), and 10 mM glutamate (G) (Sigma Aldrich) were added in order to activate complex I. OXPHOS capacity was recorded at saturating concentrations of 2.5 mM ADP (Sigma Aldrich) after the addition of 10 mM succinate (S) (Sigma Aldrich). The simultaneous presence of all substrates in the cuvette allowed the determination of the total OXPHOS activity. The uncoupled maximal ETS capacity was determined by titration with the uncoupler carbonyl cyanide 3-chlorophenylhydrazone, CCCP (Sigma Aldrich, 0.5 μM) up to the complete dissipation of the proton gradient. The residual oxygen consumption (ROX) respiration was achieved by addition of rotenone and antimycin (Sigma Aldrich, 2 and 2.5 μM respectively).

### 4.5. Complex II Activity Analysis

Independent activity of complex II (succinate dehydrogenase) was measured by HRR with a specific protocol modified from [39]. Permeabilized cells were treated with 2 μM rotenone in the presence of pyruvate and malate (5 and 2 mM respectively) [39]. Activity of complex II (OXPHOS sustained by complex II) was achieved by stimulation with 10 mM succinate in the presence of saturating ADP concentrations (2.5 mM). As counter-proof, the complex II inhibitor malonic acid (Sigma Aldrich, 5 mM) was added at the end of the experiment. In parallel, a control experiment performed without rotenone was carried out to achieve the total OXPHOS respiration. This state was used as a reference for normalization.

### 4.6. Data Analysis

Instrumental and chemical background fluxes were opportunely calibrated as a function of oxygen concentration using DatLab software (Oroboros Instruments). Rate of oxygen consumption in the respiratory states ROUTINE, LEAK, OXPHOS, maximal ETS capacity was corrected for the ROX. The oxygen respiratory flux was expressed as pmol/s per million cells or as FCRs calculated for each state relative to the maximal uncoupled ETS capacity (used in this work as the reference state) [34,35]. Oxygen flux coupled to ATP synthesis was determined by correcting each state for the LEAK respiration and expressed as FCRs [34,35]. Coupling efficiencies were calculated by correcting each state for LEAK respiration and expressing it as a ratio of the total capacity in that specific state, as indicated [34,35]. The activity of complex II was calculated as SCFs normalizing the oxygen flux linked to complex II for the convergent flux of complex I + complex II, both measured during OXPHOS stimulation [39].

### 4.7. Statistical Analysis

All data are expressed as means with standard deviation. At least three independent experiments were performed. Data were statistically analyzed by t-test using GraphPad Prism software. The following values * *p* < 0.05, ** *p* < 0.01, *** *p* < 0.001 were taken as significant.

## Supplementary Materials

Supplementary materials can be found at https://www.mdpi.com/1422-0067/21/21/7809/s1. Table S1. Oxygen flux calculated for each respiratory state corrected for the ROX respiration in untreated (CTRL) and MPP+ treated cells; Table S2. FCR calculated for each respiratory state as ETS percentage in untreated (CTRL) and MPP+ treated cells; Table S3. FCR calculated for net and coupling respiration and E-R capacity factor.

## Abbreviations

PD	Parkinson’s disease
ND	Neurodegenerative disease
DA	Dopamine
LBs	Lewy’s bodies
MPTP	1-methyl-4-phenyl-1,2,3,6-tetrahydropyridine
MPP^+^	1-methyl-4-phenylpyridinium ion
ETS	Electron transport system
HRR	High-resolution respirometry
RA	Retinoic acid
TH	Tyrosine hydroxylase
SUIT	Substrate-uncoupler-inhibitor titration
FCR	Flux control ratio
SCF	Substrate control factor
IMM	Inner mitochondrial membrane
OMM	Outer mitochondrial membrane

## References

[1] Kalia L.V., Lang A.E. (2015). Parkinson’s disease. Lancet.

[2] Kim W.S., Kagedal K., Halliday G.M. (2014). Alpha-synuclein biology in Lewy body diseases. Alzheimer’s Res. Ther..

[3] Marchetti B., Leggio L., L’Episcopo F., Vivarelli S., Tirolo C., Paternò G., Giachino C., Caniglia S., Serapide M.F., Iraci N. (2020). Glia-Derived Extracellular Vesicles in Parkinson’s Disease. J. Clin. Med..

[4] Hirsch E.C., Jenner P., Przedborski S. (2013). Pathogenesis of Parkinson’s disease. Mov. Disord..

[5] Leggio L., Patern G., Vivarelli S., Episcopo F.L., Tirolo C., Raciti G., Pappalardo F., Giachino C., Caniglia S., Serapide M.F. (2020). Extracellular Vesicles as Nanotherapeutics for Parkinson ’s Disease. Biomolecules.

[6] Leggio L., Arrabito G., Ferrara V., Vivarelli S., Paternò G., Marchetti B., Pignataro B., Iraci N. (2020). Mastering the Tools: Natural versus Artificial Vesicles in Nanomedicine. Adv. Healthc. Mater..

[7] Bandres-Ciga S., Diez-Fairen M., Kim J.J., Singleton A.B. (2020). Genetics of Parkinson’s disease: An introspection of its journey towards precision medicine. Neurobiol. Dis..

[8] Marchetti B. (2020). Nrf2/Wnt resilience orchestrates rejuvenation of glia-neuron dialogue in Parkinson’s disease. Redox Biol..

[9] Toffoli M., Vieira S.R.L., Schapira A.H.V. (2020). Genetic causes of PD: A pathway to disease modification. Neuropharmacology.

[10] Marchetti B. (2018). Wnt/β-catenin signaling pathway governs a full program for dopaminergic neuron survival, neurorescue and regeneration in the MPTP mouse model of Parkinson’s disease. Int. J. Mol. Sci..

[11] William Langston J., Ballard P., Tetrud J.W., Irwin I. (1983). Chronic parkinsonism in humans due to a product of meperidine-analog synthesis. Science.

[12] Langston J.W., Forno L.S., Tetrud J., Reeves A.G., Kaplan J.A., Karluk D. (1999). Evidence of active nerve cell degeneration in the substantia nigra of humans years after 1-methyl-4-phenyl-1,2,3,6-tetrahydropyridine exposure. Ann. Neurol..

[13] Jackson-Lewis V., Przedborski S. (2007). Protocol for the MPTP mouse model of Parkinson’s disease. Nat. Protoc..

[14] Langston J.W. (2017). The MPTP story. J. Parkinsons. Dis..

[15] Baltazar M.T., Dinis-Oliveira R.J., de Lourdes Bastos M., Tsatsakis A.M., Duarte J.A., Carvalho F. (2014). Pesticides exposure as etiological factors of Parkinson’s disease and other neurodegenerative diseases-A mechanistic approach. Toxicol. Lett..

[16] Vaccari C., El Dib R., de Camargo J.L.V. (2017). Paraquat and Parkinson’s disease: A systematic review protocol according to the OHAT approach for hazard identification. Syst. Rev..

[17] Maiti P., Manna J., Dunbar G.L., Maiti P., Dunbar G.L. (2017). Current understanding of the molecular mechanisms in Parkinson’s disease: Targets for potential treatments. Transl. Neurodegener..

[18] Di Monte D.A., Wu E.Y., Irwin I., Delanney L.E., Langston J.W. (1991). Biotransformation of 1-methyl-4-phenyl-1,2,3,6-tetrahydropyridine in primary cultures of mouse astrocytes. J. Pharmacol. Exp. Ther..

[19] Heikkila R.E., Hess A., Duvoisin R.C. (1984). Dopaminergic neurotoxicity of 1-methyl-4-phenyl-1,2,5,6-tetrahydropyridine in mice. Science.

[20] Javitch J.A., D’Amato R.J., Strittmatter S.M., Snyder S.H. (1985). Parkinsonism-inducing neurotoxin, N-methyl-4-phenyl-1,2,3,6-tetrahydropyridine: Uptake of the metabolite N-methyl-4-phenylpyridine by dopamine neurons explains selective toxicity. Proc. Natl. Acad. Sci. USA.

[21] Watanabe Y., Himeda T., Araki T. (2005). Mechanisms of MPTP toxicity and their implications for therapy of Parkinson’s disease. Med. Sci. Monit..

[22] Nicklas W.J., Vyas I., Heikkila R.E. (1985). Inhibition of NADH-linked oxidation in brain mitochondria by 1-methyl-4-phenyl-pyridine, a metabolite of the neurotoxin, 1-methyl-4-phenyl-1,2,5,6-tetrahydropyridine. Life Sci..

[23] Bose A., Beal M.F. (2016). Mitochondrial dysfunction in Parkinson’s disease. J. Neurochem..

[24] Mapa M.S.T., Le V.Q., Wimalasena K. (2018). Characteristics of the mitochondrial and cellular uptake of MPP+, as probed by the fluorescent mimic, 4’I-MPP+. PLoS ONE.

[25] Zhu J.H., Gusdon A.M., Cimen H., Van Houten B., Koc E., Chu C.T. (2012). Impaired mitochondrial biogenesis contributes to depletion of functional mitochondria in chronic MPP+ toxicity: Dual roles for ERK1/2. Cell Death Dis..

[26] Choi W.S., Kruse S.E., Palmiter R.D., Xia Z. (2008). Mitochondrial complex I inhibition is not required for dopaminergic neuron death induced by rotenone, MPP+, or paraquat. Proc. Natl. Acad. Sci. USA.

[27] Biedler J.L., Schachner M. (1978). Multiple Neurotransmitter Synthesis by Human Neuroblastoma Cell Lines and Clones. Cancer Res..

[28] Ross R.A., Biedler J.L. (1985). Presence and Regulation of Tyrosinase Activity in Human Neuroblastoma Cell Variants in Vitro. Cancer Res..

[29] Påhlman S., Ruusala A.I., Abrahamsson L., Mattsson M.E.K., Esscher T. (1984). Retinoic acid-induced differentiation of cultured human neuroblastoma cells: A comparison with phorbolester-induced differentiation. Cell. Differ..

[30] Xicoy H., Wieringa B., Martens G.J.M. (2017). The SH-SY5Y cell line in Parkinson’s disease research: A systematic review. Mol. Neurodegener..

[31] Lopes F.M., Schröder R., Júnior M.L.C. (2010). da F.; Zanotto-Filho, A.; Müller, C.B.; Pires, A.S.; Meurer, R.T.; Colpo, G.D.; Gelain, D.P.; Kapczinski, F.; et al. Comparison between proliferative and neuron-like SH-SY5Y cells as an in vitro model for Parkinson disease studies. Brain Res..

[32] Cheung Y.T., Lau W.K.W., Yu M.S., Lai C.S.W., Yeung S.C., So K.F., Chang R.C.C. (2009). Effects of all-trans-retinoic acid on human SH-SY5Y neuroblastoma as in vitro model in neurotoxicity research. Neurotoxicology.

[33] Teppola H., Sarkanen J.R., Jalonen T.O., Linne M.L. (2016). Morphological Differentiation Towards Neuronal Phenotype of SH-SY5Y Neuroblastoma Cells by Estradiol, Retinoic Acid and Cholesterol. Neurochem. Res..

[34] Pesta D., Gnaiger E. (2012). High-resolution respirometry: OXPHOS protocols for human cells and permeabilized fibers from small biopsies of human muscle. Methods Mol. Biol..

[35] Gnaiger E., MitoEAGLE Task Group (2020). Mitochondrial physiology. Bioenerg. Commun..

[36] Zhu J.H., Horbinski C., Guo F., Watkins S., Uchiyama Y., Chu C.T. (2007). Regulation of autophagy by extracellular signal-regulated protein kinases during 1-methyl-4-phenylpyridinium-induced cell death. Am. J. Pathol..

[37] Zilocchi M., Finzi G., Lualdi M., Sessa F., Fasano M., Alberio T. (2018). Mitochondrial alterations in Parkinson’s disease human samples and cellular models. Neurochem. Int..

[38] Dukes A.A., Bai Q., Van Laar V.S., Zhou Y., Ilin V., David C.N., Agim Z.S., Bonkowsky J.L., Cannon J.R., Watkins S.C. (2016). Live imaging of mitochondrial dynamics in CNS dopaminergic neurons in vivo demonstrates early reversal of mitochondrial transport following MPP+ exposure. Neurobiol. Dis..

[39] Gnaiger E. (2009). Capacity of oxidative phosphorylation in human skeletal muscle. New perspectives of mitochondrial physiology. Int. J. Biochem. Cell Biol..

[40] Djafarzadeh S., Jakob S.M. (2017). High-resolution respirometry to assess mitochondrial function in permeabilized and intact cells. J. Vis. Exp..

[41] Pfleger J., He M., Abdellatif M. (2015). Mitochondrial complex II is a source of the reserve respiratory capacity that is regulated by metabolic sensors and promotes cell survival. Cell Death Dis..

[42] Evinova A., Cizmarova B., Hatokova Z., Racay P. (2020). High-Resolution Respirometry in Assessment of Mitochondrial Function in Neuroblastoma SH-SY5Y Intact Cells. J. Membr. Biol..

[43] Stepanova A., Shurubor Y., Valsecchi F., Manfredi G., Galkin A. (2016). Differential susceptibility of mitochondrial complex II to inhibition by oxaloacetate in brain and heart. Biochim. Biophys. Acta Bioenerg..

[44] Manczak M., Reddy P.H. (2012). Abnormal interaction of VDAC1 with amyloid beta and phosphorylated tau causes mitochondrial dysfunction in Alzheimer’s disease. Hum. Mol. Genet..

[45] Smilansky A., Dangoor L., Nakdimon I., Ben-Hail D., Mizrachi D., Shoshan-Barmatz V. (2015). The voltage-dependent anion channel 1 mediates amyloid β toxicity and represents a potential target for Alzheimer disease therapy. J. Biol. Chem..

[46] Israelson A., Arbel N., Da Cruz S., Ilieva H., Yamanaka K., Shoshan-Barmatz V., Cleveland D.W. (2010). Misfolded mutant SOD1 directly inhibits VDAC1 conductance in a mouse model of inherited ALS. Neuron.

[47] Magrì A., Belfiore R., Reina S., Tomasello M.F., Di Rosa M.C., Guarino F., Leggio L., De Pinto V., Messina A. (2016). Hexokinase i N-terminal based peptide prevents the VDAC1-SOD1 G93A interaction and re-establishes ALS cell viability. Sci. Rep..

[48] Shteinfer-Kuzmine A., Argueti S., Gupta R., Shvil N., Abu-Hamad S., Gropper Y., Hoeber J., Magrì A., Messina A., Kozlova E.N. (2019). A VDAC1-Derived N-Terminal Peptide Inhibits Mutant SOD1-VDAC1 Interactions and Toxicity in the SOD1 Model of ALS. Front. Cell. Neurosci..

[49] Rostovtseva T.K., Gurnev P.A., Protchenko O., Hoogerheide D.P., Yap T.L., Philpott C.C., Lee J.C., Bezrukov S.M. (2015). α-synuclein shows high affinity interaction with voltage-dependent anion channel, suggesting mechanisms of mitochondrial regulation and toxicity in Parkinson disease. J. Biol. Chem..

[50] Magri A., Messina A. (2017). Interactions of VDAC with proteins involved in neurodegenerative aggregation: An opportunity for advancement on therapeutic molecules. Curr. Med. Chem..

[51] Leggio L., Guarino F., Magrì A., Accardi-Gheit R., Reina S., Specchia V., Damiano F., Tomasello M.F., Tommasino M., Messina A. (2018). Mechanism of translation control of the alternative Drosophila melanogaster Voltage Dependent Anion-selective Channel 1 mRNAs. Sci. Rep..

[52] Magrì A., Di Rosa M.C., Tomasello M.F., Guarino F., Reina S., Messina A., De Pinto V. (2016). Overexpression of human SOD1 in VDAC1-less yeast restores mitochondrial functionality modulating beta-barrel outer membrane protein genes. Biochim. Biophys. Acta Bioenerg..

[53] Messina A., Reina S., Guarino F., De Pinto V. (2012). VDAC isoforms in mammals. Biochim. Biophys. Acta Biomembr..

[54] Magrì A., Di Rosa M.C., Orlandi I., Guarino F., Reina S., Guarnaccia M., Morello G., Spampinato A., Cavallaro S., Messina A. (2020). Deletion of Voltage-Dependent Anion Channel 1 knocks mitochondria down triggering metabolic rewiring in yeast. Cell. Mol. Life Sci..

[55] Hoogerheide D.P., Gurnev P.A., Rostovtseva T.K., Bezrukov S.M. (2017). Mechanism of α-synuclein translocation through a VDAC nanopore revealed by energy landscape modeling of escape time distributions. Nanoscale.

[56] Devi L., Raghavendran V., Prabhu B.M., Avadhani N.G., Anandatheerthavarada H.K. (2008). Mitochondrial import and accumulation of α-synuclein impair complex I in human dopaminergic neuronal cultures and Parkinson disease brain. J. Biol. Chem..

[57] Elkon H., Don J., Melamed E., Ziv I., Shirvan A., Offen D. (2002). Mutant and wild-type α-synuclein interact with mitochondrial cytochrome C oxidase. J. Mol. Neurosci..

[58] Fritz R.R., Abell C.W., Patel N.T., Gessner W., Brossi A. (1985). Metabolism of the neurotoxin in MPTP by human liver monoamine oxidase B. FEBS Lett..

[59] Jastroch M., Divakaruni A.S., Mookerjee S., Treberg J.R., Brand M.D. (2010). Mitochondrial proton and electron leaks. Essays Biochem..

[60] Ramonet D., Perier C., Recasens A., Dehay B., Bové J., Costa V., Scorrano L., Vila M. (2013). Optic atrophy 1 mediates mitochondria remodeling and dopaminergic neurodegeneration linked to complex i deficiency. Cell Death Differ..

[61] Calabria E., Scambi I., Bonafede R., Schiaffino L., Peroni D., Potrich V., Capelli C., Schena F., Mariotti R. (2019). Ascs-exosomes recover coupling efficiency and mitochondrial membrane potential in an in vitro model of als. Front. Neurosci..

[62] Ricquier D., Bouillaud F. (2000). The uncoupling protein homologues: UCP1, UCP2, UCP3, StUCP and AtUCP. Biochem. J..

[63] Porter R.K. (2001). Mitochondrial proton leak: A role for uncoupling proteins 2 and 3?. Biochim. Biophys. Acta Bioenerg..

[64] Cannon B., Shabalina I.G., Kramarova T.V., Petrovic N., Nedergaard J. (2006). Uncoupling proteins: A role in protection against reactive oxygen species-or not?. Biochim. Biophys. Acta Bioenerg..

[65] Ho P.W.L., Chan D.Y.L., Kwok K.H.H., Chu A.C.Y., Ho J.W.M., Kung M.H.W., Ramsden D.B., Ho S.L. (2005). Methyl-4-phenylpyridinium ion modulates expression of mitochondrial uncoupling proteins 2, 4, and 5 in catecholaminergic (SK-N-SH) cells. J. Neurosci. Res..

[66] Chu A.C.Y., Ho P.W.L., Kwok K.H.H., Ho J.W.M., Chan K.H., Liu H.F., Kung M.H.W., Ramsden D.B., Ho S.L. (2009). Mitochondrial UCP4 attenuates MPP+- and dopamine-induced oxidative stress, mitochondrial depolarization, and ATP deficiency in neurons and is interlinked with UCP2 expression. Free Radic. Biol. Med..

[67] Kwok K.H.H., Ho P.W.L., Chu A.C.Y., Ho J.W.M., Liu H.F., Yiu D.C.W., Chan K.H., Kung M.H.W., Ramsden D.B., Ho S.L. (2010). Mitochondrial UCP5 is neuroprotective by preserving mitochondrial membrane potential, ATP levels, and reducing oxidative stress in MPP+ and dopamine toxicity. Free Radic. Biol. Med..

[68] Ho P.W.L., Ho J.W.M., Tse H.M., So D.H.F., Yiu D.C.W., Liu H.F., Chan K.H., Kung M.H.W., Ramsden D.B., Ho S.L. (2012). Uncoupling protein-4 (UCP4) increases ATP supply by interacting with mitochondrial complex II in neuroblastoma cells. PLoS ONE.

[69] L’Episcopo F., Serapide M.F., Tirolo C., Testa N., Caniglia S., Morale M.C., Pluchino S., Marchetti B. (2011). A Wnt1 regulated Frizzled-1/β-catenin signaling pathway as a candidate regulatory circuit controlling mesencephalic dopaminergic neuron-astrocyte crosstalk: Therapeutical relevance for neuron survival and neuroprotection. Mol. Neurodegener..

[70] Shipley M.M., Mangold C.A., Szpara M.L. (2016). Differentiation of the SH-SY5Y human neuroblastoma cell line. J. Vis. Exp..

