# Supplementary material for: High-Resolution Respirometry Reveals MPP+ Mitochondrial Toxicity Mechanism in a Cellular Model of Parkinson’s Disease
Source: Int J Mol Sci. 2020 Oct 22;21(21):7809. doi: 10.3390/ijms21217809 (PMC7659480; doi:10.3390/ijms21217809)
Supplement: Supplementary file 1 [file ijms-21-07809-s001.pdf]

Supplemental Materials

Table S1. Oxygen flux calculated for each respiratory state corrected for the ROX respiration in untreated (CTRL) and MPP^+ treated cells. Data are reported as mean ± standard deviation of $n = 5$ independent experiments.

	CTRL	*MPP⁺*
ROUTINE	15.75 ± 3.68	4.77 ± 2.14
LEAK	5.62 ± 0.95	3.64 ± 1.86
OXPHOS sustained by complex I	18.74 ± 4.28	5.68 ± 1.60
OXPHOS sustained by complex I & II	27.57 ± 5.97	10.15 ± 2.16
ETS	30.27 ± 5.49	11.29 ± 2.24
ETS sustained by complex II	12.96 ± 2.41	7.99 ± 2.71

Table S2. FCR calculated for each respiratory state as ETS percentage in untreated (CTRL) and MPP^+ treated cells. Data are reported as mean ± standard deviation of $n = 5$ independent experiments.

	CTRL	*MPP^+*
ROUTINE	0.51 ± 0.03	0.41 ± 0.12
LEAK	0.19 ± 0.05	0.31 ± 0.09
OXPHOS sustained by complex I	0.61 ± 0.03	0.50 ± 0.13
OXPHOS sustained by complex I & II	0.90 ± 0.03	0.90 ± 0.07
ETS sustained by complex II	0.42 ± 0.02	0.76 ± 0.18

Table S3. FCR calculated for net and coupling respiration. and E–R capacity factor. All data are expressed as ETS percentage in untreated (CTRL) and MPP+ treated cells. Data are reported as mean ± standard deviation of $n = 5$ independent experiments.

	CTRL	*MPP^+*
netROUTINE control ratio	0.32 ± 0.05	0.09 ± 0.07
netOXPOS control ratio	0.71 ± 0.08	0.58 ± 0.12
ROUTINE coupling efficiency	0.62 ± 0.10	0.23 ± 0.15
OXPHOS coupling efficiency	0.78 ± 0.06	0.65 ± 0.11
OXPHOS complex I coupling efficiency	0.69 ± 0.08	0.53 ± 0.08
ETS coupling efficiency	0.80 ± 0.05	0.68 ± 0.09
E-R capacity factor	0.48 ± 0.03	0.58 ± 0.12
